# Median survival following geriatric hip fracture among 17,868 males from the Veterans Health Administration

**DOI:** 10.3389/fsurg.2023.1090680

**Published:** 2023-03-22

**Authors:** Alexander Lee, Ianto Lin Xi, Jaimo Ahn, Joseph Bernstein

**Affiliations:** ^1^ Department of Orthopaedic Surgery, University of Pennsylvania, Philadelphia, PA, United States; ^2^ Department of Orthopaedic Surgery, University of Michigan, Ann Arbor, MI, United States; ^3^ Department of Veterans Affairs Medical Center, Lieutenant Colonel Charles S. Kettles Veterans Affairs Medical Center, Ann Arbor, MI, United States; ^4^ Department of Veterans Affairs Medical Center, Corporal Michael J. Crescenz Veterans Affairs Medical Center, Philadelphia, PA, United States

**Keywords:** hip fracture, mortality, femoral neck fracture, total hip arthroplasty, surgery, hemiarthroplasty, medical decision making, prognosis

## Abstract

**Background:**

The expected value of treatments for geriatric femoral neck fracture is influenced by the predicted duration of survival after injury. Specifically, total hip arthroplasty is more suited for patients likely to live long enough to reap its longer-term benefits. For predicting short- and medium-term survival, there are many tools available, but for longer-term survival prognosis the current literature is insufficient. Our hypothesis is that patient age at the time of injury correlates with median life expectancy and survival rates, and these values can anchor a prediction regarding a given patient’s life expectancy. We therefore sought to determine median and fractional survival rates at 30 days, and 1, 2, 5 and 10 years after surgery for a large cohort of elderly patients with hip fracture as a function of age.

**Methods:**

17,868 male patients, 65–89 years of age, treated surgically for hip fracture within the Veterans Affairs system were assessed. From this set, 10,000 patients were randomly selected, and their ages at surgery and death (if any) were recorded at least 10 years post-operatively. Median and fractional survival rates were recorded at 1 month and 1, 2, 5, and 10 years. The mathematical relationship between age and median survival was determined. All findings from the 10,000-patient cohort were compared to corresponding values of the remaining 7,868 patients, to assess the predictive power of the initial observations.

**Results:**

The median survival rate for the entire cohort was 2.2 years, with 90.4% of the group surviving at 30 days. The percentage of the cohort surviving at 1, 2, 5 and 10 years after treatment was 64.5%, 52.3%, 27.1% and 8.9% respectively. Median survival was approximately (13 − (0.13 × *age-at-time-of-surgery*) years for patients of all ages.

**Conclusions:**

Median survival after geriatric hip fracture can be accurately predicted by the patient’s age at the time of injury. Median survival and fractional survival at key milestones can help estimate life-expectancy and thereby help guide treatment.

## Introduction

The choice among treatments for femoral neck fracture in geriatric patients is influenced by the predicted duration of survival after injury. At one end of the spectrum, patients unlikely to survive more than a few weeks must be identified and considered for non-operative treatment (1). At the other extreme, patients likely to live for many years must be identified as well. In such patients, total hip arthroplasty may be preferable to hemiarthroplasty, to minimize the risk of surgical revision should the patient outlive the prosthetic device (2). In short, it is necessary for orthopedic surgeons caring for geriatric patients with hip fracture to make predictions about patients’ likely life expectancy.

For short- and medium-term survival prognosis, there are many tools available (3, 4). For long-term survival, there is much less. Indeed, there are no studies guiding patient-specific survival prognosis five years after injury (5), a reasonable time span with regards to the surgery treatment options question. We therefore sought to determine the median survival, and fractional rates of survival at key milestones (30 days, and 1, 2, 5 and 10 years), as function of age, for geriatric patients with hip fractures.

To be sure, population-based statistics such as medians and fractional survival rates can only initiate the process of predicting an individual’s life expectancy. Patient-specific factors can and must also be used, to allow clinicians to “update their priors” and refine their predictions according to their best judgment. Nonetheless, life expectancy estimates are more likely to be accurate if the process begins from reliable base-case evidence.

## Methods

This study was a retrospective analysis of patients surgically treated for hip fracture in the Veterans Affairs healthcare system between January, 2000 and December, 2010. Date of death, if any, was recorded in August 2021, representing a minimum follow-up of 10.5 years. Data were obtained from the VA Informatics and Computing Infrastructure (VINCI), which is able to access the electronic medical record of all patients within the VA system.

We collected information on all male patients between 65 and 89 years of age inclusive who had a hip fracture surgically treated within the Veterans Affairs system. Subjects were identified by Current Procedural Terminology (CPT) codes 27235 (percutaneous skeletal fixation of femoral neck fracture); 27236 (open treatment of femoral neck fracture); 27244 (treatment of peri-trochanteric femoral fracture; with plate/screw) and 27245 (treatment of peri-trochanteric femoral fracture; with intramedullary implant). In addition, patients with CPT codes 27125 (hemiarthroplasty, hip) and 27130, (total hip arthroplasty) were also included, provided they were associated with Diagnosis-related group (DRG) codes signifying fracture (820.x). A total of 17,868 patients were identified.

For every patient, race, age at surgery, and age at death (if any) were recorded, from which the duration of survival in days was calculated. In addition, the location of the fracture was inferred from the CPT code: codes 27235, 27236, 27125 and 27130 signified femoral neck fractures, whereas 27244 and 27245 identified the peri-trochanteric fractures.

VINCI provided the relevant information from the VA Corporate Data Warehouse, which ensures that no patients are lost to follow-up.

Using a random number generator, our entire cohort of 17,868 patients was split into two groups: a cohort of 10,000 on which all analyses were performed (designated the “RULE” group), and a group of the remaining 7,868, on which the predictive validity of all analyses were tested (designated the “TEST” group).

We compared the mean age at presentation and median survival of patients with femoral neck fractures with the corresponding data of patients with peri-trochanteric fractures, to determine the reasonableness of analyzing patients with both fracture types together in a single group. We articulated arbitrarily, before examination of any data, that differences of 0.5 years are to be deemed clinically meaningful and would necessitate separate analyses by fracture type.

Fractional survival rates for all patients of each year of age at surgery, 65–89, were recorded at points 1 month, 1 year, 2 years, 5 years and 10 years after treatment. Median and interquartile survival for every one-year age bin was recorded as well.

All medians and fractional survival rates were additionally reported in 5-year age bins (e.g., 65–69), to allow comparisons to other published papers that reported data in this aggregated form, and not by each year of age distinctly (6).

To provide a shorthand means of recalling the median survival as a function of age, we next determined the relationship between age at surgery and the median survival with a linear regression. We assessed the goodness of fit with the *R*^2^ statistic. For ease of clinical use, we also produced a simplified version of the equation, rounding the constant and coefficient terms, and assessed the accuracy of this simplified equation to predict the median survival of patients of all ages in the test group.

Last, we assessed the accuracy of using data from the RULE group’s fractional survival rates to predict the similar outcomes seen in the TEST group.

## Results

The mean age and median survival of all patients with femoral neck fractures, 78.92 and 2.27 years, respectively, did not differ in a clinically meaningful way (applying our pre-defined standard) from those with peri-trochanteric fractures, 78.62 and 2.14 years, respectively. Thus, patients of all fracture types were included in a unified analysis.

Among the 10,000 male patients in the RULE group, the mean age at presentation was 79.5. There were 5,668 (56.7%) patients in the RULE group with femoral neck fractures and 4,332 (43.3%) with peri-trochanteric fractures. In the TEST group, the mean age at presentation was 79.6 years. There were 4,596 (58.4%) femoral neck fractures and 3,272 (41.6%) peri-trochanteric fractures.

Among the 10,000 patients in the RULE group, there were 6,602 identified as White. There were 2,550 patients whose race was unknown, more than triple the number identified as Black, 761 patients in all. Because of the high rate of unknown race, no further analysis on this variable was conducted.

The median survival rate for the entire RULE group cohort was 2.2 years (*I*–*Q* range: 0.4–5.4 years), with 90.4% of the group surviving at 30 days. The percentage of the entire group surviving at 1, 2, 5 and 10 years after treatment was 64.5%, 52.3%, 27.1% and 8.9% respectively.

The median survival rates for each age bin in the RULE group is shown in Table 1. The fractional survival at each of the predetermined milestones for each age bin of the RULE group is shown in Table 2. The aggregated data, sorted by 5-year age bins, is shown in Table 3.

**Table 1:** Survival by age, RULE cohort.

Age bin	Number of patients (% of *n* = 10,000 cohort)	Number of patients still alive at study’s end (% of patients in this age bin)	Median survival, years	Inter-quartile range, years
65	249 (2.5%)	31 (12.4%)	4.30	1.1–10.16
66	211 (2.1%)	23 (10.9%)	5.17	1.37–9.63
67	208 (2.1%)	26 (12.5%)	3.28	0.97–8.05
68	270 (2.7%)	19 (7.0%)	3.69	0.95–8.38
69	243 (2.4%)	20 (8.2%)	3.44	0.85–8.1
70	259 (2.6%)	14 (5.4%)	3.72	1.01–7.62
71	313 (3.1%)	17 (5.4%)	3.85	1.04–7.69
72	308 (3.1%)	12 (3.9%)	3.15	0.73–7.4
73	341 (3.4%)	6 (1.8%)	2.65	0.71–7.52
74	440 (4.4%)	13 (3.0%)	2.51	0.34–6.03
75	406 (4.1%)	9 (2.2%)	2.58	0.73–5.55
76	471 (4.7%)	7 (1.5%)	2.36	0.5–5.71
77	474 (4.7%)	9 (1.9%)	2.43	0.58–5.63
78	544 (5.4%)	10 (1.8%)	2.10	0.42–4.98
79	545 (5.5%)	11 (2.0%)	2.25	0.39–5.35
80	592 (5.9%)	9 (1.5%)	1.99	0.34–4.87
81	551 (5.5%)	5 (0.9%)	2.07	0.33–4.74
82	596 (6.0%)	4 (0.7%)	1.79	0.26–3.97
83	548 (5.5%)	4 (0.7%)	1.91	0.32–4.36
84	552 (5.5%)	5 (0.9%)	1.81	0.27–4.26
85	522 (5.2%)	2 (0.4%)	1.23	0.23–3.77
86	427 (4.3%)	6 (1.4%)	1.68	0.23–4.13
87	373 (3.7%)	2 (0.5%)	1.37	0.19–3.34
88	316 (3.2%)	1 (0.3%)	1.46	0.28–3.64
89	241 (2.4%)	2 (0.8%)	1.21	0.18–3.36

**Table 2:** Fractional survival by age in the RULE cohort, *n* = 10,000.

Age bin	Fractional survival at 30 days	Fractional survival at 1 years	Fractional survival at 2 years	Fractional survival at 5 years	Fractional survival at 10 years
65	96%	76%	66%	47%	26%
66	96%	80%	69%	52%	23%
67	96%	75%	62%	38%	21%
68	94%	74%	62%	42%	20%
69	95%	73%	63%	37%	18%
70	93%	75%	64%	41%	17%
71	94%	76%	65%	41%	17%
72	92%	71%	58%	39%	17%
73	93%	69%	57%	35%	16%
74	89%	65%	55%	32%	12%
75	93%	72%	57%	29%	10%
76	93%	65%	54%	29%	9%
77	92%	66%	56%	29%	9%
78	91%	67%	52%	25%	6%
79	88%	61%	52%	28%	7%
80	90%	61%	50%	24%	6%
81	90%	62%	51%	23%	5%
82	87%	61%	47%	19%	4%
83	90%	61%	48%	21%	4%
84	89%	61%	48%	20%	3%
85	86%	54%	40%	17%	3%
86	88%	56%	47%	19%	4%
87	85%	57%	39%	14%	1%
88	87%	57%	43%	16%	2%
89	84%	53%	40%	15%	2%

**Table 3:** Median and fractional survival by age, aggregated in 5-year age bins, of rule cohort.

Age cohort	Number of patients (%)	Median survival (with *I*–*Q* range)	% of the group surviving at day 30	% of the group surviving at 1 year	% of the group surviving at year 2	% of the group surviving at year 5	% of the group surviving at year 10
All Ages	10,000 (100%)	2.2 (0.4–5.4)	90.4%	64.5%	52.3%	27.1%	8.9%
65–69	1,181 (11.8%)	3.9 (1.0–8.7)	95.3%	75.2%	64.3%	43.1%	21.5%
70–74	1,661 (16.6%)	3.0 (0.7–6.9)	92.1%	70.4%	59.3%	36.7%	15.4%
75–79	2,440 (24.4%)	2.3 (0.5–5.3)	91.4%	65.9%	53.9%	27.7%	8.2%
80–84	2,839 (28.4%)	1.8 (0.3–4.3)	89.2%	61.2%	48.6%	21.4%	4.4%
85–90	1,879 (18.8%)	1.3 (0.2–3.7)	86.3%	55.4%	41.9%	16.5%	2.6%

There were 267 patients in the RULE group still alive at the study’s end. Almost half of this group of enduring survivors (133) were 70 years of age or younger at the time of entry into the study.

The best-fit relationship between age at the time of surgery and the median survival was described by the linear regression equation: Survival = 12.595 – 0.1303 × Age. The *R*^2^ value was 0.87. A simplified version of the equation was created by rounding: Survival = 13 – 0.13 × Age.

The simplified linear regression equation was seen to predict the median survival in the test group within one year for every age-bin category (Table 4). The mean accuracy across all 25 observations was ±0.48 years.

**Table 4:** Accuracy of the simplified equation, survival = 13 – (0.13 × Age).

Age bin	Years of predicted survival based on the simplified linear regression equation	Observed median survival, test group (years)	Absolute difference
65	4.55	4.79	0.24
66	4.42	4.42	0.00
67	4.29	4.06	0.23
68	4.16	3.95	0.21
69	4.03	3.46	0.57
70	3.90	4.03	0.13
71	3.77	3.3	0.47
72	3.64	2.92	0.72
73	3.51	2.94	0.57
74	3.38	2.64	0.74
75	3.25	2.45	0.80
76	3.12	2.55	0.57
77	2.99	2.55	0.44
78	2.86	1.86	1.00
79	2.73	1.96	0.77
80	2.60	2.00	0.60
81	2.47	1.79	0.68
82	2.34	1.95	0.39
83	2.21	1.43	0.78
84	2.08	1.66	0.42
85	1.95	1.72	0.23
86	1.82	1.68	0.14
87	1.69	1.54	0.15
88	1.56	0.83	0.73
89	1.43	1.10	0.33

The differences between the fractional survival rates of the RULE and TEST groups were assessed at each of the predetermined milestones, for each age bin. As shown in Table 5, the RULE group and TEST group data were within 10% points for all but two of the observations (lone exceptions: the one- and two-year fractional survival rates for patients exactly 88 years of age) with a mean difference for these 125 comparisons of 2.4% points.

**Table 5:** Absolute differences in fraction survival rates at post-operative milestones, comparing TEST and RULE groups in all age bins^a^.

Age bin	Fractional survival at 30 days	Fractional survival at 1 years	Fractional survival at 2 years	Fractional survival at 5 years	Fractional survival at 10 years
65	**2** **.** **6%**	*0* *.* *5%*	*1* *.* *0%*	*0* *.* *9%*	**0** **.** **4%**
66	**2** **.** **8%**	**3** **.** **1%**	**1** **.** **0%**	**9** **.** **0%**	**0** **.** **3%**
67	**1** **.** **7%**	**0** **.** **4%**	*4* *.* *7%*	*7* *.* *7%*	**0** **.** **7%**
68	*1* *.* *9%*	**2** **.** **3%**	*1* *.* *5%*	*0* *.* *1%*	**0** **.** **4%**
69	*0* *.* *7%*	**0** **.** **7%**	**1** **.** **1%**	*1* *.* *6%*	**1** **.** **1%**
70	**1** **.** **8%**	*1* *.* *9%*	*3* *.* *9%*	**3** **.** **0%**	*3* *.* *4%*
71	*0* *.* *3%*	*0* *.* *5%*	**1** **.** **7%**	**3** **.** **0%**	**2** **.** **3%**
72	*2* *.* *6%*	*4* *.* *8%*	*2* *.* *1%*	**3** **.** **0%**	**3** **.** **6%**
73	*0* *.* *6%*	*0* *.* *9%*	*2* *.* *8%*	**0** **.** **7%**	**4** **.** **2%**
74	*3* *.* *9%*	*4* *.* *7%*	*3* *.* *2%*	**0** **.** **1%**	**0** **.** **1%**
75	**1** **.** **3%**	**6** **.** **7%**	**5** **.** **0%**	*0* *.* *2%*	**2** **.** **4%**
76	**1** **.** **6%**	*3* *.* *9%*	*3* *.* *0%*	*1* *.* *4%*	**0** **.** **9%**
77	**2** **.** **6%**	*1* *.* *1%*	*0* *.* *8%*	*1* *.* *2%*	**0** **.** **7%**
78	**3** **.** **7%**	**2** **.** **8%**	**4** **.** **1%**	**3** **.** **0%**	*0* *.* *7%*
79	*1* *.* *1%*	*2* *.* *9%*	**2** **.** **8%**	**3** **.** **3%**	**0** **.** **8%**
80	*0* *.* *5%*	*2* *.* *1%*	0.0%	*1* *.* *4%*	**0** **.** **0%**
81	*0* *.* *8%*	**1** **.** **2%**	**5** **.** **0%**	**1** **.** **5%**	*0* *.* *1%*
82	*1* *.* *8%*	*3* *.* *8%*	*2* *.* *6%*	*5* *.* *2%*	*0* *.* *7%*
83	**4** **.** **4%**	**2** **.** **9%**	**3** **.** **2%**	**1** **.** **6%**	*0* *.* *1%*
84	*0* *.* *5%*	**0** **.** **8%**	**2** **.** **0%**	**2** **.** **4%**	**0** **.** **6%**
85	*1* *.* *3%*	*6* *.* *1%*	*6* *.* *0%*	*1* *.* *9%*	**1** **.** **1%**
86	**0** **.** **3%**	*4* *.* *1%*	**0** **.** **9%**	**3** **.** **0%**	**0** **.** **7%**
87	**0** **.** **0%**	**2** **.** **0%**	*4* *.* *0%*	*1* *.* *5%*	**1** **.** **0%**
88	**0** **.** **5%**	**10** **.** **4%**	**12** **.** **3%**	**4** **.** **7%**	**0** **.** **8%**
89	**0** **.** **5%**	**1** **.** **4%**	**2** **.** **1%**	**1** **.** **3%**	**2** **.** **5%**

^a^ Bold denotes that the Rule group rate was higher than that of the Test group; italic denotes that the Rule group was lower.

## Discussion

Total hip arthroplasty for femoral neck fracture is thought to be a more complex and costlier procedure than hemiarthroplasty, but is also thought to provide more long-term benefit. As such, total hip arthroplasty is more aptly suited for patients likely to live longer. Nonetheless, there is little published evidence about survival of geriatric hip fracture patients beyond two years– and at that point, the expected values of total hip arthroplasty and hemiarthroplasty might not have yet diverged (7). To fill that gap, we studied the survival patterns of a large cohort of geriatric hip fracture patients over a 10 year period.

In our study, median survival rate overall was 2.2 years, with about 10% of patients dying within 30 days and about 35% percent within a year. On the other hand, a substantial segment of this geriatric fracture population survived the injury for many years. As shown, more than one-third of patients younger than 80 lived 5 years or more, for example. We further demonstrated that median survival can be accurately predicted in males with a simple equation, Survival = 13 – 0.13 × Age, and that fractional survival rates at various time-milestones are valid prognostic markers.

It is critical that these findings be used appropriately. Median survival is only the starting point of any prognosis assessment. Indeed, by definition, half of a cohort will survive longer than the median, and half will survive less (with not necessary any subject surviving exactly this amount). If surgeons use the median as the definitive value for all patients, they are open to committing the so-called ecological fallacy, namely, drawing an unwarranted inference about specific individuals by attributing to them the aggregate value for the group. To that point, we note in passing that the age-specific median indeed failed to predict the observed survival to within 3 years for ∼30% of the individuals of the TEST group.

Despite this, the median can help initiate more accurate analyses, based on particular circumstances for a given individual. Further, the fractional rates of survival can also be used to inform decision making. For example, the finding that only 20% of 80-year-olds survive more than five years reminds us that predicted survival of that duration in such a patient faces long odds– and should be justified with additional information such as the absence of comorbidities, or a family history of longevity, for instance.

There are additional limitations of the study to be acknowledged as well. First, this was a study of male patients only. Hip fracture survival is likely to be different for females (8), and thus the results here cannot be extrapolated to both sexes. (Although geriatric hip fracture is more common in females overall, there are simply too few female military veterans to perform the analyses as conducted here.) Also, VA patients are known to have “large differences in sociodemographic status health status” (9) as compared to general patient population, and the quality of care at VA hospitals might differ from that at civilian hospitals (10). Thus, the results here might not fully extrapolate to the general population in the United States, to say nothing of residents of other countries.

The study is also somewhat limited in that some patients were still alive after the end of the study data collection period. This data censoring was rare: more than 97% of subjects had died by the study’s end. Still, the absence of a date-of-death for some patients precludes the calculation of mean and other values that rely on a complete data set. (This small amount of data censoring had no effect on the determination of medians and survival rates.)

As noted, race was not recorded in more than 25% of patients, and therefore analysis on this important variable was not possible.

This study also examined only patients indicated for surgery, a source of possible selection bias. As such, our results should be applied only to patients considered for surgical treatment. The results here likely overstate the life expectancy of all patients with hip fracture, as surgery is presumably offered only to those healthy enough to tolerate an operation.

Further, in order to study long-term survival in a large number of patients, the data collected included cases dating back more than 20 years. Prevention protocols, variations in demographics, and possible improvements in treatment and post-operative management may render some of the data here somewhat stale. The magnitude of this unavoidable effect is unknown.

An unweighted regression equation we used –to say nothing of an equation with its coefficients rounded, as we did– is less accurate. To that, we note that the regression was performed not to discover an underlying relationship but simply to create a rule of thumb that would be easily recalled. A second benefit is more philosophical. Contemplation of our equation in contrast to the perhaps more well-known estimate for the general population– that life expectancy is about 25% of (100-Age)– points out a deep truth: that geriatric hip fracture is a deadly disease, costing its sufferers about half of normal life expectancy. In any case, this simplified equation performed well in the RULE/TEST comparison.

The study might be criticized for using only one clinical variable: namely, patient age. It seems naïve to say that an accurate life expectancy prediction for a given person can be made with only one variable, and naïve it is. On the other hand, this is not what is claimed. Rather, we assert only that age can be used to predict the *group* median, an assertion that was validated. It should go without saying –but we reiterate here–that age alone is no basis for predicting an *individual’s* life expectancy. To the contrary: a group median only sets the base-case rate when deriving a more refined individual prognosis. The refinement will likely employ patient-specific factors such as the American Society of Anesthesiologists physical status classification category, scores on Charlson Comorbidity, Elixhauser, or Health-related Quality of Life Comorbidity indices, past medical history, family history, circumstances of injury, time delay to treatment, physical exam findings, mental status, functional status, preoperative lab values, hospital characteristics, surgeon characteristics, and perhaps others.

In the absence of research showing exactly how these other variables interact, the age-specific group median can be used to create an initial estimate, revised up or down according to a gestalt sense of the patient’s health (using these other variables, or others). This estimate can be checked by considering it in context of the inter-quartile ranges and fractional survival rates. We note that some estimate is needed in clinical practice, and contend that use of the survival data here improves the estimation process relative to other alternatives (a pure gestalt guesstimate, or consultation of life expectancy tables for the general population, say).

Despite these limitations, the findings here advance our knowledge of survival following geriatric hip fracture. As long as they are not used to define Procrustean rules, our results can serve as an important anchor for an estimate of life expectancy after hip fracture. Additional work is needed to validate rules for more defining the prognosis more precisely.

## Data Availability

The data analyzed in this study will be provided upon request to the corresponding author. Requests to access these datasets should be directed to orthodoc@post.harvard.edu.
